# Deregulated expression of a longevity gene, *Klotho*, in the *C9orf72* deletion mice with impaired synaptic plasticity and adult hippocampal neurogenesis

**DOI:** 10.1186/s40478-020-01030-4

**Published:** 2020-09-04

**Authors:** Wan Yun Ho, Sheeja Navakkode, Fujia Liu, Tuck Wah Soong, Shuo-Chien Ling

**Affiliations:** 1 Department of Physiology, National University of Singapore, Singapore, 117549 Singapore; 2 Lee Kong Chian School of Medicine, Nanyang Technological University, Singapore, 636921 Singapore; 3 Centre for Healthy Longevity, National University Health System, Singapore, Singapore; 4 Program in Neuroscience and Behavior Disorders, Duke-NUS Medical School, Singapore, 169857 Singapore; 5 Department of Physiology, Yong Loo Lin School of Medicine, National University of Singapore, Tahir Foundation Building, MD1, 16-03-H, 12 Science Drive 2, Singapore, 117549 Singapore

**Keywords:** Amyotrophic lateral sclerosis (ALS), Frontotemporal dementia (FTD), C9ORF72, Klotho, Longevity, Dentate gyrus, adult neurogenesis, Long-term potentiation (LTP), Long-term depression (LTD)

## Abstract

Hexanucleotide repeat expansion of *C9ORF72* is the most common genetic cause of amyotrophic lateral sclerosis and frontotemporal dementia. Synergies between loss of C9ORF72 functions and gain of toxicities from the repeat expansions contribute to C9ORF72-mediated pathogenesis. However, how loss of *C9orf72* impacts neuronal and synaptic functions remains undetermined. Here, we showed that long-term potentiation at the dentate granule cells and long-term depression at the Schaffer collateral/commissural synapses at the area CA1 were reduced in the hippocampus of *C9orf72* knockout mice. Using unbiased transcriptomic analysis, we identified that *Klotho*, a longevity gene, was selectively dysregulated in an age-dependent manner. Specifically, Klotho protein expression in the hippocampus of *C9orf72* knockout mice was incorrectly enriched in the dendritic regions of CA1 with concomitant reduction in granule cell layer of dentate gyrus at 3-month of age followed by an accelerating decline during aging. Furthermore, adult hippocampal neurogenesis was reduced in *C9orf72* knockout mice. Taken together, our data suggest that C9ORF72 is required for synaptic plasticity and adult neurogenesis in the hippocampus and Klotho deregulations may be part of C9ORF72-mediated toxicity.

**Electronic supplementary material:**

The online version of this article (10.1186/s40478-020-01030-4) contains supplementary material, which is available to authorized users.

Hexanucleotide repeat expansion of *C9ORF72* is the most frequent genetic cause of amyotrophic lateral sclerosis (ALS) and frontotemporal dementia (FTD) [10, 33]. Although loss of *C9orf72* does not cause neurodegeneration per se [5, 18, 21, 31], reduced *C9orf72* expression exacerbates the gain of toxicities inflicted by the repeat expansion [36, 37, 43]. Specifically, loss of *C9orf72* triggers systemic and neuronal inflammation [5, 18, 31], in part, through altering gut microbiota [6]. Molecularly, C9ORF72 acts as GDP/GTP exchange factors (GEFs) for several small RAB GTPases that are potentially involved in membrane trafficking [1, 35, 40, 41]. Furthermore, we and others have showed that C9ORF72 associates with ULK1-autophagy initiation complex to regulate autophagy [17, 19, 35, 38–41] and C9ORF72 is required for neuronal and dendritic morphogenesis via ULK1-mediated autophagy [17]. In addition, increased C9ORF72 expression due to intermediate repeat expansion disrupts autophagy and is associated with corticobasal degeneration [7], suggesting that varying C9ORF72 levels may evoke different pathogenic pathways. However, how C9ORF72 may contribute to neuronal and synaptic dysfunction remains to be defined.

Accumulating evidence indicate that synaptic impairment is a common and early event in major neurodegenerative diseases [16, 27, 32]. To investigate whether *C9orf72* knockout mice develop synaptic deficits, we measured the long-term potentiation (LTP) and long-term depression (LTD) in the CA1 and dentate gyrus (DG) of the hippocampus (see below). LTP and LTD, which measure the enduring changes in synaptic strength, has been used as the cellular models of synaptic plasticity for learning and memory [20, 30]. Furthermore, LTP and LTD dysfunctions typically correlate and may underlie the cognitive deficit often observed in a broad spectrum of neurological disorders [11, 27].

*C9orf72* knockout (*c9orf72*^−*/*−^*)* mice, where exon 2–6 were replaced with a neomycin and lacZ cassette, were described previously (Additional file 1: Supplemental Figure 1a) [17, 18]. *C9orf72* knockout mice showed premature lethality (Additional file 1: Supplemental Figure 1b). The shortened lifespan of *C9orf72* knockout mice has been attributed to systemic inflammation [5, 18, 31]. Consistent with these previous reports, the *C9orf72* knockout mice in our colony also have enlarged spleens (splenomegaly) (Additional file 1: Supplemental Figure 2). Thus, it is likely these mice die of auto-immune disease. Furthermore, the survival curve was similar to the Harvard group’s mice [5], but appeared to accelerate when compared with the UCSD group’s mice [18], potentially due to environmental factors [6]. Since the *C9orf72* knockout mice began to die after 100 days of age, we focused our analysis on a 3-month timepoint, where *C9orf72* knockout mice showed normal locomotor activities in the open field assay (Additional file 1: Supplemental Figure 1c).

To address synaptic dysfunctions that may be associated with loss of C9ORF72 functions, we first examined synaptic plasticity in corticohippocampal connections, where the inputs from entorhinal cortex project via the perforant path to the granule cells of dentate gyrus (DG) (Fig. 1a). We used a theta burst stimulation (TBS) protocol to induce LTP in DG by stimulating the medial perforant path as described previously [9]. After a stable baseline of 30 min in synaptic inputs S1, theta burst stimulation was applied to S1 which resulted in a stable late-LTP which lasted for the recorded time period of 3 h in wild type mice (Fig. 1b, Additional file 1: Supplemental Table 1–2). In contrast, the perforant path mediated-LTP at DG (thereafter abbreviated as DG-LTP) was reduced in *C9orf72* knockout mice (*p* < 0.05, Fig. 1c, Additional file 1: Supplemental Table 1–2).

**Fig. 1:**
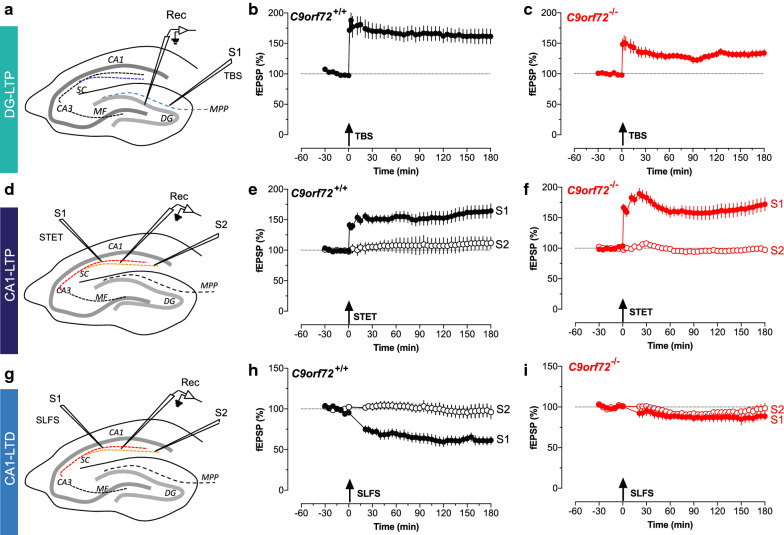
*C9orf72* knockout mice showed LTP and LTD deficits in the hippocampus. **a** Schematic of the transverse hippocampal slice showing the positioning of the electrodes in dentate gyrus (DG) area. A single stimulating electrode S1 was placed in the medial performant path input. The recording electrode was placed in the dentate granule layer and lowered to the same level to record fEPSPs. **b** Late-LTP induced by TBS in the DG shows that a stable long-lasting LTP can be recorded in DG of WT (wild type) mice by using TBS (filled circles) (n = 7). Control inputs were stable throughout the 3-h time period of recording. **c** The same experiment was repeated in *C9orf72* knockout mice, which shows that late-LTP is significantly impaired in *C9orf72* knockout mice compared to the WT (filled circles). Control inputs remained stable for 3 h (open circles) Analog traces always represent typical field EPSP traces 30 min before (dotted line), 30 min after (dashed line) and 3 h (solid line) after tetanization. Arrows indicate the time point of induction of plasticity in the corresponding synaptic input. **d** Schematics of the transverse hippocampal slice showing the positioning of the electrodes in hippocampal area CA1. The two independent synaptic inputs S1 and S2 to the same neuronal population and the recording sites (rec) for the field EPSPs are shown. **e** The time course of fEPSP after the induction of LTP by STET in 3-month-old WT littermates of *C9orf72* knockout mice (filled circles). The open circles represent the control synaptic input S2 (n = 7). **f** Late-LTP induced by STET in *C9orf72* knockout mice at 3 months shows that the post tetanic potentiation is higher, but after 20 min the percentage of potentiation comes back to normal like late-LTP in WT littermates (n = 8). **g** Schematics of the transverse hippocampal slice showing the positioning of the electrodes. The two independent synaptic inputs S1 and S2 to the same neuronal population and the recording sites (rec) for the field EPSPs are shown. **h** Late-LTD was induced using a strong low frequency stimulation (SLFS) in S1 (filled circles) which resulted in the weakening of synaptic responses that remained stable throughout the recording period of 3 h. Control inputs remained stable throughout the recorded time period (open circles) (n = 6). **i** The time course of the fEPSPs after low frequency stimulation in *C9orf72* knockout mice at 3 months showed, the absence of LTD (filled circles) (n = 7). Open circles represent the control synaptic input (open circles). Analog traces always represent typical field EPSP traces 30 min before (dotted line), 30 min after (dashed line) and 3 h (solid line) after tetanization. Arrows indicate the time point of SLFS/tetanization of the corresponding synaptic input

For assessing synaptic plasticity of the CA1 area, we used an established long-term potentiation (LTP) paradigm by stimulating Schaffer collateral fibers that send input to the CA1 dendritic regions. After recording a stable baseline, strong tetanus stimulation (STET) was given via the stimulating electrode S1, whereas the stimulating electrode S2 served as a control for the input specificity of LTP (Fig. 1d) [34]. Under this condition, LTP was induced and maintained for more than 3 h in both wild type and *C9orf72* knockout mice at 3-month of age (Fig. 1e, f, Additional file 1: Supplemental Table 1–2). The control input S2 remained stable throughout the time of recording (Fig. 1e, f). The data indicates that LTP at the area CA1 (thereafter abbreviated as CA1-LTP) was normal in *C9orf72* knockout mice at 3 months of age.

Next, we determined whether the induction and maintenance of long-term depression (LTD) is affected in the CA1 synapses. To do so, a strong low frequency stimulation (SLFS) was delivered to the S1 input in the CA1 of hippocampus, whereas S2 served as a control (Fig. 1g). A significant depression (thereafter abbreviated as CA1-LTD) was observed and remained stable throughout the time period of recording in the wild type mice (Fig. 1h, Additional file 1: Supplemental Table 1–2). In contrast, this CA1-LTD was abolished in the *C9orf72* knockout mice (*p* < 0.01, Additional file 1: Supplemental Table 1–2), while the response to the control input S2 remained stable throughout the time period of recording (Fig. 1i). Collectively, these electrophysiological data suggest that there are deficits in the synaptic plasticity in DG and CA1 regions of hippocampus in the *C9orf72* knockout mice, where DG-LTP and CA1-LTD, but not CA1-LTP, is reduced.

To investigate how loss of *C9orf72* may be required for regulating synaptic plasticity, we performed transcriptomic analysis on the hippocampi isolated from *C9orf72* knockout mice and their wild type littermate controls at 3 months of age using Affymetrix GeneChip mouse microarray that covers coding and noncoding RNAs (Fig. 2a). Using a 2-fold-change cut-off, there are 48 up-regulated genes and 12 down-regulated genes. 14 of 48 (29.1%) up- and 7 of 12 (58.3%) down-regulated genes belong to the noncoding RNAs (Fig. 2b). Gene ontology (GO) analysis of these differentially expressed genes (DEGs) revealed that they are enriched with secreted proteins and glycoproteins (Fig. 2c). The most down-regulated genes, including *C9orf72* itself, *Gm7120* and *Zfp932*, and the most up-regulated genes, including *Htr2c*, *Kl*, *Enpp2*, *Clic6*, *Kcnj*, and *Ttr*, were further validated using qRT-PCR (Additional file 1: Supplemental Figure 3).

**Fig. 2:**
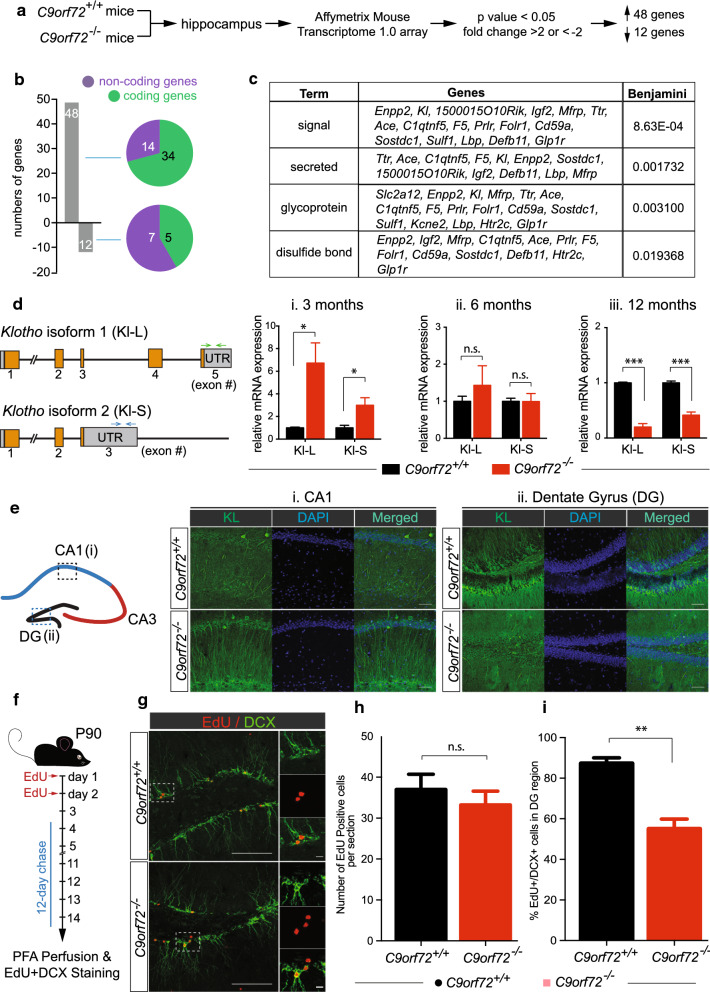
*C9orf72* regulates the expression of a longevity gene, *Klotho*, and is required for adult neurogenesis in the hippocampus. **a** Schematic for transcriptomic analysis of the hippocampus from wild type and *C9orf72* knockout mice. **b** Total of 60 differentially expressed genes (DEGs) were identified. Among them, 48 and 12 genes are up- and down-regulated, respectively. Furthermore, 14 of 48 (29.1%) up- and 7 of 12 (58.3%) down-regulated genes belong to the noncoding RNAs (magenta). **c** Gene otology analysis of up-regulated DEGs. **d** Age-dependent deregulation of Klotho expression in *C9orf72* knockout mice. Schematic of two Klotho isoforms due to the alternative usage of exon 3. RNAs were extracted from wild type and *C9orf72* knockout mice, reverse transcribed and quantified using primers specific for isoform 1 and 2 of Klotho gene. Sub-panel i, ii, and iii are qRT-PCR results for 3, 6, and 12-month animals. *p < 0.05, ***p < 0.0001. (di) 3 months, KL-L: *p* = 0.0328; KL-S, *p* = 0.0476, (dii) 6 months, KL-L: *p* = 0.4734; KL-S, *p* = 0.9766, and (diii) 12 months, KL-L, *p* = 0.0002; KL-S, *p* = 0.0008. n = 3, per genotype, per timepoint. **e** Confocal images of Klotho protein in CA1 and DG region of wild type and C9orf72 knockout mice. Klotho immunoreactivity is increased at the dendritic region of CA1 and reduced in the granule cell layer of DG in the *C9orf72* knockout mice. Scale bar is 20 μm. n = 3 per genotype. **f** Schematic of EdU-pulse chase experiment (left panel). **g** Confocal image of EdU/doublecortin (DCX) staining. Scale bar is 50 and 10 μm, respectively. **h** Quantification of EdU-positive cells. **i** Quantification of EdU/DCX-double positive cells in the DG region. (3–5 slices per animals, n = 3 per genotype, *p* < 0.05)

Among these DEGs, *Kl* (encodes Klotho) is of particular interest. *KLOTHO* has been proposed to be a longevity gene, where whole body deletion of *Klotho* in mice causes accelerated aging and premature death [23] and systemic over-expressing *Klotho* enhances cognition and extend lifespan [12, 24]. Mouse *Kl* can be alternatively spliced to give rise to a membrane bound form (isoform 1, Kl-L) and secreted form (isoform 2, Kl-S) (Fig. 2d). Using primers that are specific to isoform 1 and isoform 2, we further confirmed that both *Kl* isoforms were increased to 6- (*p* < 0.05) and 3.5-fold (*p* < 0.05), respectively, in the hippocampi of *C9orf72* knockout mice when compared with the wild type mice at 3 months of age (Fig. 2di). Intriguingly, the *Klotho* mRNA expressions of both isoforms became comparable at 6 months of age (Fig. 2dii) and reduced by 80% (isoform 1, *p* < 0.0001) and 60% (isoform 2, *p* < 0.0001) at 12 months of age (Fig. 2diii). The data suggest that KLOTHO levels are dysregulated in the *C9orf72* knockout mice in an age-dependent manner.

To further investigate the Klotho expression pattern in the *C9orf72* knockout mice, Klotho immunofluorescence was performed. The Klotho expression within the dendritic region of CA1 was increased (Fig. 2ei). In contrast, the Klotho expression within the granule cell layer of DG was reduced at 3 months of age (Fig. 2eii). Thus, although total Klotho expression was elevated in 3-month-old *C9orf72* knockout mice, the pattern of Klotho expression was altered. Consistent with the qRT-PCR data, Klotho levels were comparable between WT and C9orf72 knockout mice at 6 months of age (Additional file 1: Supplemental Figure 4).

As (i) varying Klotho levels affect adult neurogenesis in the hippocampus [25], and (ii) Klotho expression within DG is reduced in the *C9orf72* knockout mice, we hypothesized that adult hippocampal neurogenesis may be affected in the *C9orf72* knockout mice. To test this, we determined the rate of adult neurogenesis by performing an EdU-pulse chase experiment (Fig. 2f). EdU, a thymidine analogue that is incorporated into DNA during replication, was used to label new born cells for two constitutive days and then chased for 12 days, until the new born progenitor cells had matured into neurons [15]. The degree of neurogenesis was quantified by co-labeling EdU-positive cells with doublecortin (DCX), an immature neuronal marker (Fig. 2f, g). The total numbers of EdU-positive cells were comparable between the control and the *C9orf72* knockout mice (Fig. 2h). However, we observed a 30% of reduction (*p* < 0.05) of EdU/DCX-double positive cells in the DG region (Fig. 2i), indicating the adult hippocampal neurogenesis is reduced in the *C9orf72* knockout mice.

In this study, we showed the loss of *C9orf72* impairs DG-LTP and CA1-LTD as well as adult neurogenesis in the hippocampus. New born neurons provide additional plasticity to the brain and are involved in spatial memory, pattern separation and stress resilience [4, 14]. Furthermore, adult hippocampal neurogenesis appears to be reduced dramatically in patients with Alzheimer’s disease [29], highlighting the potential role of impaired adult neurogenesis in the pathogenesis of neurodegenerative diseases. Thus, our results suggest that defective synaptic functions and adult neurogenesis may contribute to C9ORF72-mediated pathogenesis. We further identified that a longevity gene, *Klotho*, is mis-regulated in the hippocampus of *C9orf72* knockout mice. In particular, Klotho levels are reduced in the DG, where adult neurogenesis occurs, followed by an accelerated reduction in the hippocampus at 12 months of age. Klotho is a pleiotropic protein and involved in regulating the homeostasis of phosphate, calcium, and vitamin D [22]. Although the exact function of Klotho in the central nervous system (CNS) is not known, it has been shown to enhance N-methyl-D-aspartate receptor (NMDAR)-mediated synaptic activity [12] and oligodendrocyte maturation [8]. Furthermore, Klotho has been shown to regulate hippocampal synaptic plasticity [12, 26, 28]. Thus, it is conceivable that ablation of *C9orf72* alters the Klotho expression and affects Klotho-mediated regulation on synaptic plasticity. Importantly, polymorphisms in the *KLOTHO* gene (known as KL-VS variant) have been identified to associate with a longer lifespan [2], better cognition in human [12], and is protective for the *APOE4* carriers in Alzheimer’s disease [3, 13]. In the context of ALS, overexpressing Klotho was beneficial in protecting neuronal loss in a SOD1 mouse model [42]. In conclusion, our results highlight that (1) C9ORF72 is required for synaptic plasticity and adult neurogenesis in the hippocampus, and (2) the expression of longevity gene, *Klotho*, may be one of the downstream effectors of C9ORF72 and could have implications in ALS-FTD spectrum diseases.

## Supplementary information

**Additional file 1**: Supplementary information, including detailed materials and methods and supplemental figures.

## References

[1] Aoki Y, Manzano R, Lee Y, Dafinca R, Aoki M, Douglas AGL, Varela MA, Sathyaprakash C, Scaber J, Barbagallo P, Vader P, Mäger I, Ezzat K, Turner MR, Ito N, Gasco S, Ohbayashi N, El Andaloussi S, Takeda S, Fukuda M, Talbot K, Wood MJA (2017). C9orf72 and RAB7L1 regulate vesicle trafficking in amyotrophic lateral sclerosis and frontotemporal dementia. Brain.

[2] Arking DE, Krebsova A, Macek M, Macek M, Arking A, Mian IS, Fried L, Hamosh A, Dey S, McIntosh I, Dietz HC (2002). Association of human aging with a functional variant of klotho. Proc Natl Acad Sci USA.

[3] Belloy ME, Napolioni V, Han SS, Le Guen Y, Greicius MD, For the Alzheimer’s Disease Neuroimaging Initiative (2020). Association of Klotho-VS heterozygosity with risk of Alzheimer disease in individuals who carry APOE4. JAMA Neurol.

[4] Bond AM, Ming G, Song H (2015). Adult mammalian neural stem cells and neurogenesis: five decades later. Stem Cell.

[5] Burberry A, Suzuki N, Wang J-Y, Moccia R, Mordes DA, Stewart MH, Suzuki-Uematsu S, Ghosh S, Singh A, Merkle FT, Koszka K, Li Q-Z, Zon L, Rossi DJ, Trowbridge JJ, Notarangelo LD, Eggan K (2016). Loss-of-function mutations in the C9ORF72 mouse ortholog cause fatal autoimmune disease. Sci Transl Med.

[6] Burberry A, Wells MF, Limone F, Couto A, Smith KS, Keaney J, Gillet G, van Gastel N, Wang J-Y, Pietilainen O, Qian M, Eggan P, Cantrell C, Mok J, Kadiu I, Scadden DT, Eggan K (2020). C9orf72 suppresses systemic and neural inflammation induced by gut bacteria. Nature.

[7] Cali CP, Patino M, Tai YK, Ho WY, McLean CA, Morris CM, Seeley WW, Miller BL, Gaig C, Vonsattel JPG, White CL, Roeber S, Kretzschmar H, Troncoso JC, Troakes C, Gearing M, Ghetti B, Van Deerlin VM, Lee VM-Y, Trojanowski JQ, Mok KY, Ling H, Dickson DW, Schellenberg GD, Ling S-C, Lee EB (2019). C9orf72 intermediate repeats are associated with corticobasal degeneration, increased C9orf72 expression and disruption of autophagy. Acta Neuropathol.

[8] Chen C-D, Sloane JA, Li H, Aytan N, Giannaris EL, Zeldich E, Hinman JD, Dedeoglu A, Rosene DL, Bansal R, Luebke JI, Kuro-o M, Abraham CR (2013). The antiaging protein Klotho enhances oligodendrocyte maturation and myelination of the CNS. J Neurosci.

[9] Chew B, Ryu JR, Ng T, Ma D, Dasgupta A, Neo SH, Zhao J, Zhong Z, Bichler Z, Sajikumar S, Goh ELK (2015). Lentiviral silencing of GSK-3β in adult dentate gyrus impairs contextual fear memory and synaptic plasticity. Front Behav Neurosci.

[10] DeJesus-Hernandez M, Mackenzie IR, Boeve BF, Boxer AL, Baker M, Rutherford NJ, Nicholson AM, Finch NA, Gilmer HF, Adamson J, Kouri N, Wojtas A, Sengdy P, Hsiung G-YR, Karydas A, Seeley WW, Josephs KA, Coppola G, Geschwind DH, Wszolek ZK, Feldman H, Knopman D, Petersen R, Miller BL, Dickson D, Boylan K, Graff-Radford N, Rademakers R (2011). Expanded GGGGCC hexanucleotide repeat in non-coding region of C9ORF72 causes chromosome 9p-linked frontotemporal dementia and amyotrophic lateral sclerosis. Neuron.

[11] Dong Z, Han H, Li H, Bai Y, Wang W, Tu M, Peng Y, Zhou L, He W, Wu X, Tan T, Liu M, Wu X, Zhou W, Jin W, Zhang S, Sacktor TC, Li T, Song W, Wang YT (2015). Long-term potentiation decay and memory loss are mediated by AMPAR endocytosis. J Clin Invest.

[12] Dubal DB, Yokoyama JS, Zhu L, Broestl L, Worden K, Wang D, Sturm VE, Kim D, Klein E, Yu G-Q, Ho K, Eilertson KE, Yu L, Kuro-o M, De Jager PL, Coppola G, Small GW, Bennett DA, Kramer JH, Abraham CR, Miller BL, Mucke L (2014). Life extension factor klotho enhances cognition. Cell Rep.

[13] Erickson CM, Schultz SA, Oh JM, Darst BF, Ma Y, Norton D, Betthauser T, Gallagher CL, Carlsson CM, Bendlin BB, Asthana S, Hermann BP, Sager MA, Blennow K, Zetterberg H, Engelman CD, Christian BT, Johnson SC, Dubal DB, Okonkwo OC (2019). KLOTHO heterozygosity attenuates APOE4-related amyloid burden in preclinical AD. Neurology.

[14] Gage FH (2019). Adult neurogenesis in mammals. Science.

[15] Gonçalves JT, Schafer ST, Gage FH (2016). Adult neurogenesis in the hippocampus: from stem cells to behavior. Cell.

[16] Henstridge CM, Pickett E, Spires-Jones TL (2016). Synaptic pathology: a shared mechanism in neurological disease. Ageing Res Rev.

[17] Ho WY, Tai YK, Chang J-C, Liang J, Tyan S-H, Chen S, Guan J-L, Zhou H, Shen H-M, Koo E, Ling S-C (2019). The ALS-FTD-linked gene product, C9orf72, regulates neuronal morphogenesis via autophagy. Autophagy.

[18] Jiang J, Zhu Q, Gendron TF, Saberi S, McAlonis-Downes M, Seelman A, Stauffer JE, Jafar-Nejad P, Drenner K, Schulte D, Chun S, Sun S, Ling S-C, Myers B, Engelhardt J, Katz M, Baughn M, Platoshyn O, Marsala M, Watt A, Heyser CJ, Ard MC, De Muynck L, Daughrity LM, Swing DA, Tessarollo L, Jung CJ, Delpoux A, Utzschneider DT, Hedrick SM, de Jong PJ, Edbauer D, Van Damme P, Petrucelli L, Shaw CE, Bennett CF, Da Cruz S, Ravits J, Rigo F, Cleveland DW, Lagier-Tourenne C (2016). Gain of toxicity from ALS/FTD-linked repeat expansions in C9ORF72 is alleviated by antisense oligonucleotides targeting GGGGCC-containing RNAs. Neuron.

[19] Jung J, Nayak A, Schaeffer V, Starzetz T, Kirsch AK, Müller S, Dikic I, Mittelbronn M, Behrends C (2017). Multiplex image-based autophagy RNAi screening identifies SMCR8 as ULK1 kinase activity and gene expression regulator. Elife.

[20] Kessels HW, Malinow R (2009). Synaptic AMPA receptor plasticity and behavior. Neuron.

[21] Koppers M, Blokhuis AM, Westeneng H-J, Terpstra ML, Zundel CAC, Vieira de Sá R, Schellevis RD, Waite AJ, Blake DJ, Veldink JH, van den Berg LH, Pasterkamp RJ (2015). C9orf72 ablation in mice does not cause motor neuron degeneration or motor deficits. Ann Neurol.

[22] Kuro-o M (2013). Klotho, phosphate and FGF-23 in ageing and disturbed mineral metabolism. Nat Rev Nephrol.

[23] Kuro-o M, Matsumura Y, Aizawa H, Kawaguchi H, Suga T, Utsugi T, Ohyama Y, Kurabayashi M, Kaname T, Kume E, Iwasaki H, Iida A, Shiraki-Iida T, Nishikawa S, Nagai R, Nabeshima YI (1997). Mutation of the mouse klotho gene leads to a syndrome resembling ageing. Nature.

[24] Kurosu H, Yamamoto M, Clark JD, Pastor JV, Nandi A, Gurnani P, McGuinness OP, Chikuda H, Yamaguchi M, Kawaguchi H, Shimomura I, Takayama Y, Herz J, Kahn CR, Rosenblatt KP, Kuro-o M (2005). Suppression of aging in mice by the hormone Klotho. Science.

[25] Laszczyk AM, Fox-Quick S, Vo HT, Nettles D, Pugh PC, Overstreet-Wadiche L, King GD (2017). Klotho regulates postnatal neurogenesis and protects against age-related spatial memory loss. NBA.

[26] Li D, Jing D, Liu Z, Chen Y, Huang F, Behnisch T (2019). Enhanced expression of secreted α-Klotho in the hippocampus alters nesting behavior and memory formation in mice. Front Cell Neurosci.

[27] Li Q, Navakkode S, Rothkegel M, Soong TW, Sajikumar S, Korte M (2017). Metaplasticity mechanisms restore plasticity and associativity in an animal model of Alzheimer’s disease. Proc Natl Acad Sci USA.

[28] Li Q, Vo HT, Wang J, Fox-Quick S, Dobrunz LE, King GD (2017). Klotho regulates CA1 hippocampal synaptic plasticity. Neuroscience.

[29] Moreno-Jiménez EP, Flor-García M, Terreros-Roncal J, Rábano A, Cafini F, Pallas-Bazarra N, Ávila J, Llorens-Martín M (2019). Adult hippocampal neurogenesis is abundant in neurologically healthy subjects and drops sharply in patients with Alzheimer’s disease. Nat Med.

[30] Nabavi S, Fox R, Proulx CD, Lin JY, Tsien RY, Malinow R (2014). Engineering a memory with LTD and LTP. Nature.

[31] O’Rourke JG, Bogdanik L, Yáñez A, Lall D, Wolf AJ, Muhammad AKMG, Ho R, Carmona S, Vit JP, Zarrow J, Kim KJ, Bell S, Harms MB, Miller TM, Dangler CA, Underhill DM, Goodridge HS, Lutz CM, Baloh RH (2016). C9orf72 is required for proper macrophage and microglial function in mice. Science.

[32] Picconi B, Piccoli G, Calabresi P (2012). Synaptic dysfunction in Parkinson’s disease. Adv Exp Med Biol.

[33] Renton AE, Majounie E, Waite A, Simón-Sánchez J, Rollinson S, Gibbs JR, Schymick JC, Laaksovirta H, van Swieten JC, Myllykangas L, Kalimo H, Paetau A, Abramzon Y, Remes AM, Kaganovich A, Scholz SW, Duckworth J, Ding J, Harmer DW, Hernandez DG, Johnson JO, Mok K, Ryten M, Trabzuni D, Guerreiro RJ, Orrell RW, Neal J, Murray A, Pearson J, Jansen IE, Sondervan D, Seelaar H, Blake D, Young K, Halliwell N, Callister J, Toulson G, Richardson A, Gerhard A, Snowden J, Mann D, Neary D, Nalls MA, Peuralinna T, Jansson L, Isoviita V-M, Kaivorinne A-L, Hölttä-Vuori M, Ikonen E, Sulkava R, Benatar M, Wuu J, Chiò A, Restagno G, Borghero G, Sabatelli M, Heckerman D, Rogaeva E, Zinman L, Rothstein J, Sendtner M, Drepper C, Eichler EE, Alkan C, Abdullaev Z, Pack SD, Dutra A, Pak E, Hardy J, Singleton A, Williams NM, Heutink P, Pickering-Brown S, Morris HR, Tienari PJ, Traynor BJ (2011). A hexanucleotide repeat expansion in C9ORF72 is the cause of chromosome 9p21-linked ALS-FTD. Neuron.

[34] Sajikumar S, Navakkode S, Frey JU (2005). Protein synthesis-dependent long-term functional plasticity: methods and techniques. Curr Opin Neurobiol.

[35] Sellier C, Campanari M-L, Julie Corbier C, Gaucherot A, Kolb-Cheynel I, Oulad-Abdelghani M, Ruffenach F, Page A, Ciura S, Kabashi E, Charlet-Berguerand N (2016). Loss of C9ORF72 impairs autophagy and synergizes with polyQ Ataxin-2 to induce motor neuron dysfunction and cell death. EMBO J.

[36] Shao Q, Liang C, Chang Q, Zhang W, Yang M, Chen J-F (2019). C9orf72 deficiency promotes motor deficits of a C9ALS/FTD mouse model in a dose- dependent manner. Acta Neuropathol Commun.

[37] Shi Y, Lin S, Staats KA, Li Y, Chang W-H, Hung S-T, Hendricks E, Linares GR, Wang Y, Son EY, Wen X, Kisler K, Wilkinson B, Menendez L, Sugawara T, Woolwine P, Huang M, Cowan MJ, Ge B, Koutsodendris N, Sandor KP, Komberg J, Vangoor VR, Senthilkumar K, Hennes V, Seah C, Nelson AR, Cheng T-Y, Lee S-JJ, August PR, Chen JA, Wisniewski N, Hanson-Smith V, Belgard TG, Zhang A, Coba M, Grunseich C, Ward ME, van den Berg LH, Pasterkamp RJ, Trotti D, Zlokovic BV, Ichida JK (2018). Haploinsufficiency leads to neurodegeneration in C9ORF72 ALS/FTD human induced motor neurons. Nat Med.

[38] Sullivan PM, Zhou X, Robins AM, Paushter DH, Kim D, Smolka MB, Hu F (2016). The ALS/FTLD associated protein C9orf72 associates with SMCR8 and WDR41 to regulate the autophagy-lysosome pathway. Acta Neuropathol Commun.

[39] Ugolino J, Ji YJ, Conchina K, Chu J, Nirujogi RS, Pandey A, Brady NR, Hamacher-Brady A, Wang J (2016). Loss of C9orf72 enhances autophagic activity via deregulated mTOR and TFEB signaling. PLoS Genet.

[40] Webster CP, Smith EF, Bauer CS, Moller A, Hautbergue GM, Ferraiuolo L, Myszczynska MA, Higginbottom A, Walsh MJ, Whitworth AJ, Kaspar BK, Meyer K, Shaw PJ, Grierson AJ, De Vos KJ (2016). The C9orf72 protein interacts with Rab1a and the ULK1 complex to regulate initiation of autophagy. EMBO J.

[41] Yang M, Liang C, Swaminathan K, Herrlinger S, Lai F, Shiekhattar R, Chen JF (2016). A C9ORF72/SMCR8-containing complex regulates ULK1 and plays a dual role in autophagy. Sci Adv.

[42] Zeldich E, Chen C-D, Avila R, Medicetty S, Abraham CR (2015). The anti-aging protein klotho enhances remyelination following cuprizone-induced demyelination. J Mol Neurosci.

[43] Zhu Q, Jiang J, Gendron TF, McAlonis-Downes M, Jiang L, Taylor A, Diaz Garcia S, Ghosh Dastidar S, Rodriguez MJ, King P, Zhang Y, La Spada AR, Xu H, Petrucelli L, Ravits J, Da Cruz S, Lagier-Tourenne C, Cleveland DW (2020). Reduced C9ORF72 function exacerbates gain of toxicity from ALS/FTD-causing repeat expansion in C9orf72. Nat Neurosci.

