# Supplementary material for: Deregulated expression of a longevity gene, Klotho, in the C9orf72 deletion mice with impaired synaptic plasticity and adult hippocampal neurogenesis
Source: Acta Neuropathol Commun. 2020 Sep 4;8:155. doi: 10.1186/s40478-020-01030-4 (PMC7473815; doi:10.1186/s40478-020-01030-4)
Supplement: Supplementary file 1 — Additional file 1: Supplementary information, including detailed materials and methods and supplemental figures. [file 40478_2020_1030_MOESM1_ESM.pdf]

Supplementary information

Materials and Methods

C9orf72 knockout mice

The *C9orf72* knockout mice were generated as described previously [2, 3] and maintained on a C57BL/6J background. *C9orf72*$^{+/-}$ mice were crossed with *C9orf72*$^{+/-}$ or *C9orf72*$^{-/-}$ mice to generate *C9orf72*$^{+/+}$, *C9orf72*$^{+/-}$, and *C9orf72*$^{-/-}$ mice. Only *C9orf72*$^{+/+}$ and *C9orf72*$^{-/-}$ mice were used for this study. All experiments were carried out under the protocols approved by the Institutional Animal Care and Use Committee of the National University of Singapore, and were in compliance with Association for Assessment of Laboratory Animal Care guidelines for animal use. For genotyping, genomic DNA was isolated from tail biopsies using salt extraction methods and subjected to routine PCR methods using the following primers:

C9orf72-null: 5'- ctatgaaaggttgggcttcg-3' (neomycin) and 5'- gtgggagggtatgggaaact-3' (mouse *C9orf72*)

Mouse *C9orf72:* 5'- gaatggagatcggagcacttatgg-3' and 5'- gggacagcagcttagttactaaggc-3'

Acute hippocampal slice electrophysiology

For the acute hippocampal slice preparation, mice were first anaesthetized using CO2 before being decapitated, and having their brains quickly removed and transferred into cold (2-4 °C), oxygenated artificial cerebrospinal fluid (ACSF). The ACSF contained the following (in mM): 124 NaCl, 4.9 KCl, 1.2 KH_2PO_4, 2.0 $MgSO_4$, 2.0 $CaCl_2$, 24.6 $NaHCO_3$, and 10 D-glucose, equilibrated with 95% O_2/5% CO_2 (32 L/h). Hippocampus was isolated and transverse hippocampal slices (400 µm) were sectioned by using a manual tissue chopper. The slices were incubated in an interface brain slice chamber (Scientific Systems Design, Mississauga, Ontario, Canada) at 32 °C for three hours at an ACSF flow rate of 0.8 mL min^{-1}.

For all of our experiments (except the recording from DG), we used a two-pathway experiment paradigm, where two monopolar stainless-steel electrodes (5 MΩ; AM Systems) were

positioned at an adequate distance within the stratum radiatum of the CA1 region for stimulating two independent synaptic inputs S1 and S2 to a single neuronal population, thus evoking field fEPSPs from Schaffer collateral/commissural-CA1 synapses. For recording the fEPSP (measured as its initial slope function), one electrode (5 MΩ; AM Systems) was placed in the CA1 apical dendritic layer. For dentate gyrus (DG), one monopolar lacquer-coated, stainless-steel electrode (5 MΩ; AM Systems, United States of America) was placed in the stratum moleculare of the DG to stimulate the medial perforant path input. About 200 µm apart, the recording electrode was lowered to the same level to record fEPSPs. The signals were amplified using a differential amplifier (Model 1700; AM Systems) and digitized by using a CED 1401 analog-to-digital converter (Cambridge Electronic Design).

After 3-hour of pre-incubation, an input-output curve (I-O curve), which uses afferent stimulation vs. fEPSP slope, was taken. Test stimulation intensity was adjusted to elicit fEPSP slope of 40% of the maximal EPSP response for synaptic inputs S1 and S2. Late-LTP was induced using three repeated high frequency stimulus trains of 100 pulses ("strong" tetanus (STET), 100 Hz; duration, 0.2 ms/polarity; intertrain intervals, 10 min). Late-LTP was induced in DG by a single theta burst stimulation (TBS) protocol paradigm consisting of 15 bursts of eight pulses, 200 Hz, interburst interval 200 ms. Late-LTD was induced in CA1 area using a strong low-frequency stimulation (SLFS) protocol of 900 bursts [one burst consisted of three stimuli at 20 Hz, and the interburst interval was 1 s (i.e., f = 1 Hz; stimulus duration, 0.2 ms/half wave; total number of stimuli, 2700)]. This stimulation pattern produced a stable late-LTD *in vitro* for 3 h. The slopes of the fEPSPs were monitored online. Four 0.2-Hz biphasic constant-current pulses (0.1 ms per polarity) were used for baseline recording at each time point. In all experiments, a stable baseline was recorded for at least 30 min.

Statistics

The average values of the slope function of the field EPSP (millivolts per milliseconds) per time point were analyzed using the Wilcoxon signed rank test when compared within one

group, or the Mann-Whitney U-test when data were compared between groups; $p < 0.05$ was considered as statistically significantly different. The nonparametric test was used because the analyses of the prolonged recordings do not allow the use of parametric tests.

Immunofluorescence

Tissue preparation for immunohistochemistry was described previously [1, 4]. In brief, mice were anesthetized with isoflurane and perfused transcardially with phosphate buffered saline (PBS), followed by 4% paraformaldehyde (PFA) in phosphate buffer for fixation. Brains were dissected and post-fixed in 4% PFA in PBS for 2 hours. Tissues were cryopreserved in 30% sucrose for over 24 hours and embedded in Tissue-Tek before sectioning. Brains were sectioned coronally into PBS at 30 μm using a cryostat or microtome.

For staining of hippocampal sections, sections were treated with 10 mM glycine for 15 minutes each to remove any possible PFA remnants after three washes with 1xPBS. The tissues were then permeabilized with 0.3% Triton X-100 in 1X PBS, and blocked in blocking serum, 5% donkey serum with 0.3% Triton X-100 in 1X PBS. Incubation with primary antibodies diluted in 1% donkey serum and 1X PBS in blocking serum were performed overnight at 4℃. Tissues were then washed 3 times for 15 minutes in 1X PBS. Incubation of secondary antibodies conjugated with Alexa Fluor 488, 568, 643 (1:1000, Thermo Fisher Scientific) and 1 μg/ml DAPI were done in 0.1% Triton X-100 with 1% Donkey Serum in 1X PBS for overnight at 4ºC. Tissues were washed in 0.1% Triton X-100 in 1X PBS for 3 times for 15 minutes before mounting onto slides with Prolong Gold anti-fade reagent (Thermo Fisher Scientific, P36930). The primary antibodies used in this study were: rabbit polyclonal Klotho (1:400, Abnova, PAB19446), rabbit polyclonal Doublecortin (1:800, Cell Signaling, 4604).

EdU (5-ethynyl-2'deoxyuridine) injection and staining

EdU injection and staining was described previously with modifications [4]. EdU powder (Toronto Research Chemical, #T-E932175) was dissolved in sterile 1x PBS at the stock concentration of 2.5 mg/ml. It was further diluted to 1 mg/ml in sterile 1x PBS as working concentration. Mice at P90 were injected with EdU at a dosage of 50 mg/kg intraperitoneally for 2 consecutive days. Twelve day after the last injection, mice were perfused with 1x PBS, followed by 4% PFA in phosphate buffer, brain and spinal cord were dissected for further tissue sectioning. Brains were sectioned at 30 μm thickness for EdU staining. EdU staining was performed on spinal cord sections using EdU staining solution containing 100mM Tris pH 7.5, 4mM CuSO4, 1mg/ml Sulfo-Cyanide Azide and 100mM Sodium Ascorbate in milli-Q water. Brain sections were incubated in reaction cocktail for 1 hour at room temperature in dark, followed by 3 times wash with 1x PBS. To combine with cell markers staining, sections were permeabilized with 0.3% PBST for 15 minutes, followed by blocking with blocking buffer (5% BSA, 0.5% Tween-20 in 1x PBS) for 1 hour at room temperature, before applying primary antibody overnight in dark. The following steps are the same as normal immunofluorescent staining. EdU signal was visualized using red fluorescence at 594 nm and cell markers were visualized using green fluorescence at 488 nm.

Image acquisition

Confocal images were acquired with a Zeiss LSM700 inverted confocal microscope with 4 laser lines (405/488/555/639 nm) with either a 20x/0.8 N.A. air or 63x/1.15 N.A. oil immersion objectives. Images were captured using a AxioCam MRm monochromatic CCD camera (Zeiss) run by Zeiss Zen software.

Statistics

For data with multiple measures from individual mice were observed (e.g. Fig 2H and 2I with n = 3 per genotype and 5-6 sections per mouse), the variance was fit using a linear mixed-effects models with genotype as a fixed effect and random effect of individual mice variation

using the lme4 R package [5]. The model with the lowest AICc score is selected [6] and further pairwise contrasts were performed using Tukey's HSD test from the emmeans R package with Kenward-Roger approximations of degrees of freedom [7].

RNA isolation and qRT-PCR

Total RNAs were extracted from hippocampi using Trizol reagent (Thermo Fisher Scientific) as described previously [1] according to manufacturer's instruction. After DNase treatment using RQ1 RNase-Free DNase (Promega), 1 µg RNA were reversely transcribed using Maxima First Strand cDNA Synthesis Kit for RT-qPCR (Thermo Fisher Scientific). All qRT-PCR reactions were performed with at least three biological replicates for each group and two technical replicates. mRNA levels were determined using Maxima SYBR Green qPCR master mix (Thermo Fisher Scientific). The forward and reverse primer sequences were based on pre-validated primers from PrimerBank (https://pga.mgh.harvard.edu/primerbank/) and further validated in-house. Primers for genes of interest were listed in Supplemental Table 3. Expression values were normalized to two control genes: HRPT and GAPDH mRNA. Expression values were expressed as a percentage of the average expression of control samples.

Microarray analysis

RNA quality was measured using the Agilent Bioanalyzer system. Samples with RIN (RNA integrity numbers) larger than 8.5 were used for microarray analysis according to the manufacturer's protocol. In brief, total RNAs (n=3 for each genotype) were reverse transcribed to produce cDNA/mRNA hybrid, which was subsequently used as a template to create double stranded cDNA. This double-stranded cDNA was then amplified via *in vitro* transcription to produce cRNA. *In vitro* transcription generated cRNA was then purified and subjected to 2nd-cylce single-stranded sense cDNA synthesis, which was later fragmented, labeled, and hybridized to the GeneChip Mouse Transcriptome 1.0 Array for 16 hours at 45^{0}C. Arrays were then washed, stained and scanned using an Affymetrix 3000 7G scanner. Differentially

expressed genes were selected based on $p < 0.05$ and fold-change more than 2-fold increased or decreased. Microarray data have been deposited in NCBI's Gene Expression Omnibus.

Supplemental Figure 1. Reduced survival of *C9orf72* knockout mice. (a) Schematic of genomic structure for endogenous mouse *C9orf72* and null *C9orf72* allele. Exon 2-6 was replaced with a gene-trap cassette. (b) Survival curve wild type and *C9orf72* knockout mice. (c) Open field test for wild type and *C9orf72* knockout mice at 3 months of age (n=15 for wild type mice; n=27 for *C9orf72* knockout mice).

Supplemental Figure 2. *C9orf72* knockout mice develop progress splenomegaly. Spleens from wild type (C9orf72$^{+/+}$) and *C9orf72* knockout (C9orf72$^{-/-}$) mice at 3-, 6-month-old and old-age (>12 months). Spleens from *C9orf72* knockout mice showed progressive enlargement.

Supplemental Figure 3. qRT-PCR confirmation of differentially expressed genes identified by microarray. (a) qRT-PCR validation of selective down-regulated genes include *C9orf72*, *Gm7120* and *Zfp932*. (b) qRT-PCR validation of selective up-regulated genes includes *Htr2c*, *Kl*, *Enpp2*, *Clic6*, *Kcnj*, and *Ttr*.

Supplemental Figure 4. Confocal images of Klotho protein in CA1 and DG region of wild type and *C9orf72* knockout mice at 6 months of age. KL (Klotho) is labeled in green and nuclei marker (DAPI) in blue.

Reference

1. Ho WY, Chang J-C, Tyan S-H, Yen Y-C, Lim K, Tan BSY, Ong J, Tucker-Kellogg G, Wong P, Koo E, Ling S-C (2019) FUS-mediated dysregulation of Sema5a, an autism-related gene, in FUS mice with hippocampus-dependent cognitive deficits. Human Molecular Genetics 28:3777–3791. doi: 10.1093/hmg/ddz217

2. Ho WY, Tai YK, Chang J-C, Liang J, Tyan S-H, Chen S, Guan J-L, Zhou H, Shen H-M, Koo E, Ling S-C (2019) The ALS-FTD-linked gene product, C9orf72, regulates neuronal morphogenesis via autophagy. Autophagy 15:827–842. doi: 10.1080/15548627.2019.1569441

3. Jiang J, Zhu Q, Gendron TF, Saberi S, McAlonis-Downes M, Seelman A, Stauffer JE, Jafar-Nejad P, Drenner K, Schulte D, Chun S, Sun S, Ling S-C, Myers B, Engelhardt J, Katz M, Baughn M, Platoshyn O, Marsala M, Watt A, Heyser CJ, Ard MC, De Muynck L, Daughrity LM, Swing DA, Tessarollo L, Jung CJ, Delpoux A, Utzschneider DT, Hedrick SM, de Jong PJ, Edbauer D, Van Damme P, Petrucelli L, Shaw CE, Bennett CF, Da Cruz S, Ravits J, Rigo F, Cleveland DW, Lagier-Tourenne C (2016) Gain of Toxicity from ALS/FTD-Linked Repeat Expansions in C9ORF72 Is Alleviated by Antisense Oligonucleotides Targeting GGGGCC-Containing RNAs. Neuron 90:535–550. doi: 10.1016/j.neuron.2016.04.006

4. Wang J, Ho WY, Lim K, Feng J, Tucker-Kellogg G, Nave K-A, Ling S-C (2018) Cell-autonomous requirement of TDP-43, an ALS/FTD signature protein, for oligodendrocyte survival and myelination. Proc Natl Acad Sci USA 115:E10941–E10950. doi: 10.1073/pnas.1809821115

5. D. Bates, M. Mächler, B. Bolker, S. Walker, Fitting Linear Mixed-Effects Models Using lme4. *J. Stat. Softw.* **67**, 1–48 (2015).

6. M. J. M. and portions of code contributed by D. Linden, *AICcmodavg: Model Selection and Multimodel Inference Based on (Q)AIC(c)* (2019) (April 4, 2020).

7. R. Lenth, H. Singmann, J. Love, P. Buerkner, M. Herve, *emmeans: Estimated Marginal Means, aka Least-Squares Means* (2020) (April 4, 2020).

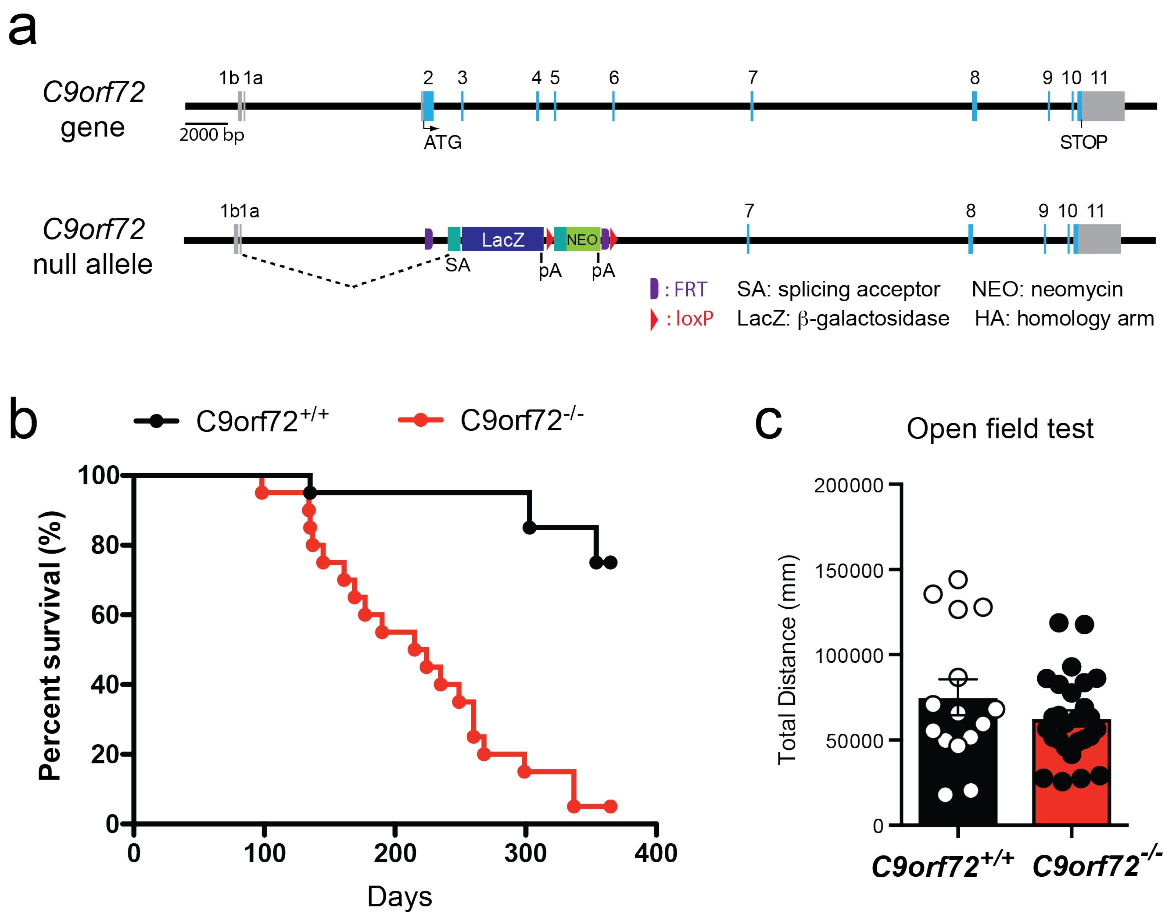

Supplemental Figure 1. Reduced survival of *C9orf72* knockout mice. (a) Schematic of genomic structure for endogenous mouse *C9orf72* and null *C9orf72* allele. Exon 2-6 was replaced with a gene-trap cassette. (b) Survival curve wild type and *C9orf72* knockout mice. (c) Open field test for wild type and *C9orf72* knockout mice at 3 months of age (n=15 for wild type mice; n=27 for *C9orf72* knockout mice).

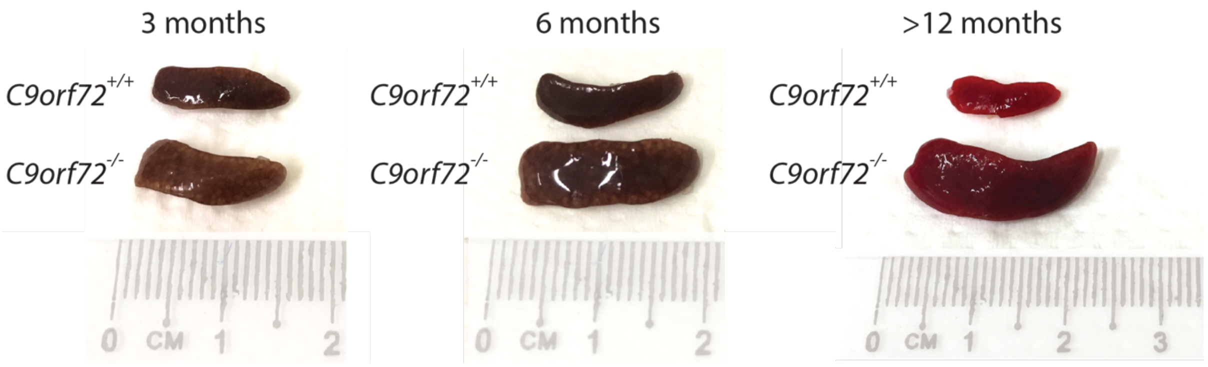

Supplemental Figure 2. *C9orf72* knockout mice develop progress splenomegaly. Spleens from wild type (C9orf72$^{+/+}$) and *C9orf72* knockout (C9orf72$^{-/-}$) mice at 3-, 6-month-old and old-age (>12 months). Spleens from *C9orf72* knockout mice showed progressive enlargement.

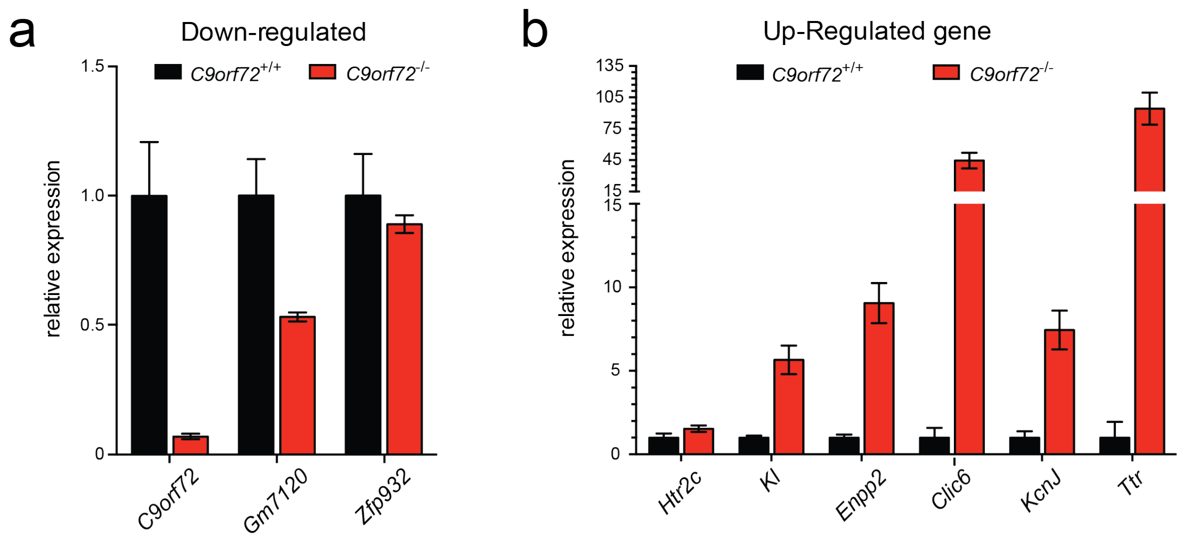

Supplemental Figure 3. qRT-PCR confirmation of differentially expressed genes identified by microarray. (a) qRT-PCR validation of selective down-regulated genes include *C9orf72*, *Gm7120* and *Zfp932*. (b) qRT-PCR validation of selective up-regulated genes includes *Htr2c*, *Kl*, *Enpp2*, *Clic6*, *Kcnj*, and *Ttr*.

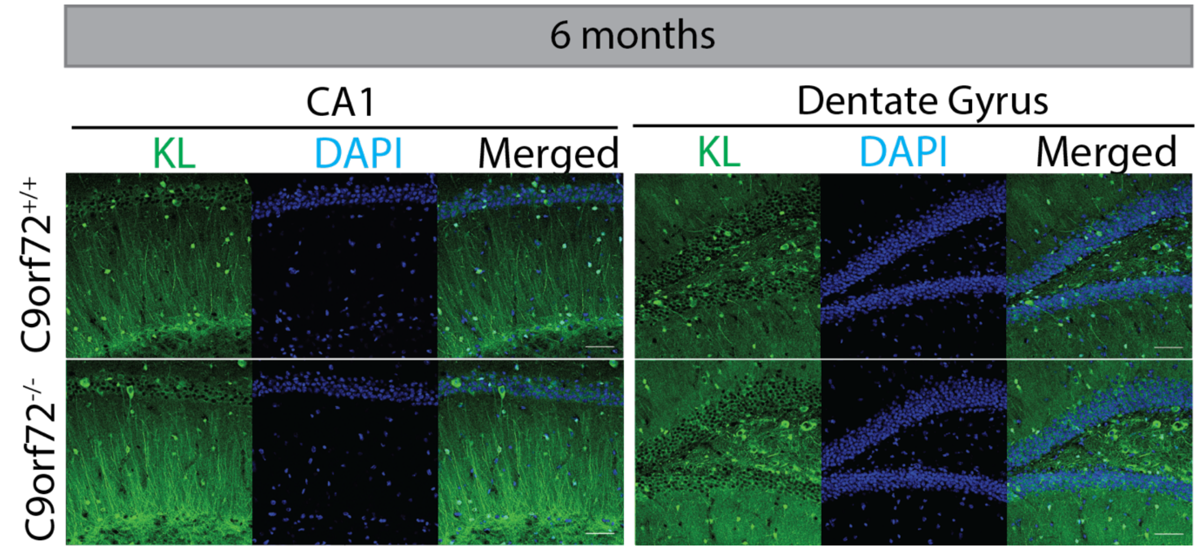

Supplemental Figure 4. Confocal images of Klotho protein in CA1 and DG region of wild type and *C9orf72* knockout mice at 6 months of age. KL (Klotho) is labeled in green and nuclei marker (DAPI) in blue.

Supplemental Table 1. Statistic analysis of DG-LTP, CA1-LTP, and CA1-LTD in wild type (WT) and C9orf72 knockout (C9KO) mice. *P* values of statistical comparisons between pre-tetanization time points and post-tetanization time points (30 min, 60 min, 120 min, and 180 min; Wilcoxon test) and the statistical comparisons with its own control groups when applicable (at 30 min, 60 min, 120 min, and 180 min; U-test).

Figure	30 min		60 min		120 min		180 min	
	Wilcoxon	U-test	Wilcoxon	U-test	Wilcoxon	U-test	Wilcoxon	U-test
1b DG-LTP WT	0.0313		0.0313		0.0313		0.0313	
1c DG-LTP C9KO	0.0313		0.0313		0.0313		0.0313	
1e CA1-LTP WT	0.0039	P<0.0001	0.0039	P<0.0001	0.0039	0.0005	0.0039	P<0.0001
1f CA1-LTP C9KO	0.0078	0.0002	0.0078	0.0002	0.0078	0.0002	0.0078	0.0002
1h CA1-LTD WT	0.0022	0.0043	0.0313	0.0043	0.0022	0.0043	0.0313	0.0152
1i CA1-LTD C9KO	0.6875	0.3829	0.1094	0.535	0.0469	0.4557	0.0313	0.535

Supplemental Table 2. Statistic analysis of DG-LTP, CA1-LTP, and CA1-LTD between wild type and *C9orf72* knockout mice. *P* values of statistical comparisons between different groups (at 30 min, 60 min, 120 min, and 180 min; U-test).

Figure	30 min<br>U-test	60 min<br>U-test	120 min<br>U-test	180 min<br>U-test
Figure 1b-c. DG-LTP<br>S1-wt/ S1-c9ko	0.1375	0.0221	0.1807	0.1014
Figure 1e-f. CA1-LTP<br>S1-wt/S1-c9ko	0.0111	0.8148	0.6058	0.4807
Figure 1h-i. CA1-LTD<br>S1-wt/S1-c9ko	0.0082	0.0513	0.0082	0.0082

Supplemental Table 3. PCR primers

**Gene Name**	**Forward Primer (5'-3')**	**Reverse Primer (5'-3')**
C9orf72	TTCCATTTGAAGCCTGGCCT	TCCCCTTCTGCGTATCATCC
Klotho (total)	GTCGACGTCATTGGGTACAC	ACAGCTCCTTGTCCTGACTC
Klotho Isoform 1 (Kl-L)	ATGCTGTCAGTGCTTGGATG	CAAGGACAGGTTCCCAGTGT
Klotho Isoform 2 (Kl-S)	GAGTGGCATAGGGGCTACAG	CAACAACTCCCCAAGCAAAG
Gm7120	GCTTTACCACCGTGCTGTAG	TGTTATCTGAGATGGCGCGA
Zfp932	TGGGCTTTGCTGAATCCTTC	TGCCTTCCATGTCTTCTGGA
Htr2c	ATCGCTGGACCGGTATGTAG	TCGTCCCTCAGTCCAATCAC
Enpp2	ACATTGAGAAACTGCGGTCC	ATTGCCAACGATTCCATGGG
Clic6	CGTCAAGGCTGGTTATGACG	AGGCTTCCGTTTTAGGTCCA
Kcnj13	GGCCTTGTGTATCTCCGAGA	ACTGCAAAGACAAGCCAGTG
Ttr	GGAGTCTGGAGAGCTGCAC	ACCACATCCGCGAATTCATG
Hprt	TCAGTCAACGGGGGACATAA	GGGGCTGTACTGCTTAACCAG
Gapdh	TCACCACCATGGAGAAGGC	GCTAAGCAGTTGGTGGTGCA

Supplemental Table 4. Statistic analysis for EdU- and EdU/DCX-positive cells in the hippocampal sections from wild type and C9orf72 knockout mice.

(1) Number of EdU+ Cells

Model ~ genotype + (1|mouseID)

Linear Mixed Model				
term	estimate	std.error	statistic	group
(Intercept)	39.15	3.37	11.61	fixed
genotype C9KO	-5.94	4.68	-1.27	fixed
sd_(Intercept) mouseID	0.00	NA	NA	mouseID
sd_Observation Residual	12.16	NA	NA	Residual

Contrasts							
level1	level2	estimate	std.error	df	statistic	p.value	signif
nontg	C9KO	5.94	4.70	1.99	1.26	0.33	-

(2) Percent of EdU+ / DCX+ cells

Model ~ genotype + (1|mouseID)

Linear Mixed Model				
term	estimate	std.error	statistic	group
(Intercept)	87.39	3.91	22.34	fixed
genotypeC9KO	-32.22	5.45	-5.91	fixed
sd_(Intercept).mouseID	0.00	NA	NA	mouseID
sd_Observation.Residual	15.65	NA	NA	Residual

Contrasts							
level1	level2	estimate	std.error	df	statistic	p.value	signif
nontg	C9KO	32.22	5.47	3.94	5.89	4.37E-03	**
